# Lenvatinib Combined with New FP Hepatic Arterial Infusion Chemotherapy for Unresectable Hepatocellular Carcinoma: Clinical Efficacy, Vascular Remodeling, and Implications for Immuno-Oncology–Systemic Combination Therapy

**DOI:** 10.3390/curroncol33050286

**Published:** 2026-05-13

**Authors:** Susumu Maruta, Yohei Koshima, Yuji Debari, Chihei Sugihara, Gou Takahata, Ryo Tamura, Tadashi Ohshima, Yuji Ono, Yuho Morita, Tomoki Chiba, Satoru Ishida, Hideto Imai, Keisuke Watanabe, Ryo Chinzei, Masanori Takahashi, Yoshihiko Ooka

**Affiliations:** 1 Department of Gastroenterology, Saitama Red Cross Hospital, 1-5 Shintoshin, Chuo-ku, Saitama 330-8553, Japan; 2 Chiba Chuo Clinic, 1-1 Honchiba-cho, Chuo-ku, Chiba 260-0014, Japan

**Keywords:** hepatocellular carcinoma, lenvatinib, hepatic arterial infusion chemotherapy, New FP, LEN-New FP, vascular remodeling, immuno-oncology

## Abstract

**Background/Objectives:** Patients with unresectable hepatocellular carcinoma (uHCC) refractory or intolerant to immune checkpoint inhibitor (ICI)-based regimens represent a growing yet therapeutically underserved population with limited treatment options. We investigated the efficacy, safety, and mechanistic underpinnings of lenvatinib combined with New FP hepatic arterial infusion chemotherapy (LEN–New FP) in this challenging clinical setting. **Methods:** We retrospectively analyzed 14 consecutive patients with uHCC treated with LEN–New FP between April 2022 and March 2025. Tumor response was assessed by the modified Response Evaluation Criteria in Solid Tumors (mRECIST). Proper hepatic artery (PHA) diameter was serially measured on angiography as an exploratory assessment of vascular remodeling, and tumor vascularity was semi-quantitatively evaluated using a 4-point angiographic scoring system (Tumor Vascularity Score [TVS]). **Results:** The cohort comprised BCLC stage B/C (7/7), mALBI grade 1–2b, and 13 of 14 patients with prior ICI-containing therapy. The objective response rate and disease control rate were 85.7% and 100%, including two complete responses. Median overall survival was 22.8 months from LEN–New FP initiation (median follow-up: 15.1 months) and 36.2 months from first-line initiation; median intrahepatic progression-free survival was 10.4 months. A total of 11 of 14 patients (78.6%) transitioned to subsequent therapies, including four curative-intent conversions. PHA narrowing was observed in 10 of 13 evaluable patients (76.9%), with no clear association with hepatic function deterioration. TVS decreased in 10 of 12 evaluable patients (83.3%), with reduction observed in 90.0% of PR/CR cases. **Conclusions:** LEN–New FP achieved sustained intrahepatic tumor control and encouraging survival in aggressive uHCC, including ICI-refractory or -intolerant disease. The concordant reduction in PHA diameter and tumor vascularity score provides angiographic evidence of VEGFR inhibition-mediated vascular remodeling, offering mechanistic insight into the synergistic antitumor effects of this regimen and supporting LEN–New FP as a promising multimodal strategy within the evolving landscape of HCC treatment.

## Simple Summary

No established treatment exists for patients with unresectable hepatocellular carcinoma (HCC) who do not respond to or cannot tolerate immune checkpoint inhibitor (ICI)-based therapy. We evaluated lenvatinib combined with New FP hepatic arterial infusion chemotherapy (LEN–New FP)—comprising intra-arterial cisplatin–lipiodol followed by continuous 5-fluorouracil infusion—in 14 patients with aggressive, ICI-refractory or -intolerant unresectable HCC. The combination achieved an objective response rate of 85.7% and a disease control rate of 100%, with a median overall survival of 22.8 months from treatment initiation. Most patients were able to proceed to subsequent therapies, including four who underwent curative-intent surgical or ablative procedures. A previously unreported finding was the narrowing of the proper hepatic artery (PHA) observed on serial angiography in the majority of patients, which appears to reflect the cooperative vascular effects of lenvatinib and intra-arterial chemotherapy and may explain the exceptionally high tumor response rate. Serial angiographic assessment of tumor vascularity using a semi-quantitative scoring system further confirmed regression of tumor-feeding vessels in the majority of patients, providing additional evidence for the vascular mechanism underlying this regimen’s antitumor activity. These findings support LEN–New FP as a promising treatment option for patients with advanced HCC after ICI failure.

## 1. Introduction

Hepatocellular carcinoma (HCC) remains a major global health challenge, representing the sixth most common cancer and the third leading cause of cancer-related mortality worldwide [1,2]. The heterogeneity of HCC, driven by differences in underlying liver disease, tumor biology, vascular invasion, and microenvironmental composition, complicates treatment and contributes to poor survival outcomes [3]. Over the past decade, systemic therapy has been fundamentally transformed by the introduction of multikinase inhibitors (e.g., sorafenib, lenvatinib) [4], and more recently, immune checkpoint inhibitors (ICIs) targeting the PD-1/PD-L1 and CTLA-4 pathways [5,6,7,8]. Combination immunotherapy regimens—including atezolizumab plus bevacizumab (IMbrave150) [5,6], durvalumab plus tremelimumab (HIMALAYA) [7], and nivolumab plus ipilimumab [8]—have produced substantial improvements in survival and have reshaped first-line treatment strategies.

However, despite these advances, a large proportion of patients remain difficult to treat [9,10,11]. A major clinical challenge is the high rate of primary ICI resistance, which is reported in approximately 30–46% of patients in real-world cohorts [10]. These patients often experience rapid disease progression within the first treatment cycles, limiting opportunities for effective subsequent sequencing. Additionally, patients with extensive intrahepatic tumor burden, bilobar involvement, or macrovascular invasion (MVI) historically have relatively poor outcomes with systemic therapy alone [4,11]. Such populations are common in East Asia but may be underrepresented in Western clinical trials [3,12,13]. Crucially, no established standard of care exists for patients who are refractory or intolerant to ICI-based first-line therapy.

In this context, locoregional therapies (LRT) play a central role in the management of HCC in Asian countries [13,14,15]. Transarterial chemoembolization (TACE), and hepatic arterial infusion chemotherapy (HAIC) remain standard options for intermediate and advanced HCC [16,17,18]. Among these, HAIC—especially the New FP regimen (intra-arterial cisplatin suspended in lipiodol followed by continuous 5-FU infusion), which is predominantly used in Japan—has demonstrated high tumor response rates even in patients with severe portal vein tumor thrombus (PVTT) [19,20,21]. Clinical trials in China further demonstrated that FOLFOX-HAIC outperformed sorafenib in advanced HCC, leading to broader international recognition of HAIC as a valuable treatment modality [22].

Simultaneously, the concept of immuno-oncology–systemic therapy integration has emerged as a key therapeutic strategy [18,23]. The TACTICS-L trial demonstrated that combining lenvatinib with TACE produced an unprecedented objective response rate approaching 90% [24]. This synergistic effect is thought to derive from vascular normalization, whereby anti-VEGF therapy remodels abnormal tumor vasculature, reducing interstitial pressure, improving oxygenation, and enhancing intratumoral distribution of intra-arterially delivered agents [25,26,27].

Lenvatinib (LEN), a potent inhibitor of VEGFR1–3, FGFR, RET, and KIT, induces early vascular remodeling [4]. In preclinical and clinical studies, LEN has been shown to normalize chaotic tumor vasculature, increase perfusion uniformity, decrease vessel permeability and leakage, suppress neovascular sprouting, and reduce the caliber of immature tumor-feeding arteries [25,26,27]. As directly demonstrated by Tachiiri et al. [28] using high-resolution DSA and perfusion CT, short-term lenvatinib administration reduces the dilatation and tortuosity of tumor-feeding vessels and decreases arterial blood flow to the tumor by approximately 30–40%, providing direct angiographic evidence of vascular normalization at the tumor neovasculature level. These biological effects provide a strong mechanistic rationale for combining LEN with HAIC. The New FP regimen delivers cisplatin at high local concentrations via lipiodol deposition and maintains prolonged 5-FU exposure through continuous infusion [20,29]. When combined with LEN-induced vascular normalization, HAIC drug delivery may become more efficient, consistent, and spatially uniform—potentially translating into deeper tumor responses [25,26].

In our previous report, we presented preliminary findings on the efficacy of LEN–New FP in a small cohort of patients with advanced HCC [21]. During the accumulation of subsequent cases, we incidentally observed narrowing of the PHA during LEN–New FP therapy on routine angiography performed for HAIC administration. Although the clinical significance of this finding remains to be fully elucidated, PHA narrowing may reflect vascular normalization, endothelial stabilization, or hemodynamic redistribution toward tumor-feeding vessels [25,26].

The present study extends our previous findings through a more comprehensive retrospective analysis, systematically evaluating survival outcomes, iPFS, hepatic reserve preservation, and vascular remodeling parameters in 14 patients with uHCC. This cohort, enriched for aggressive disease phenotypes and ICI-refractory or -intolerant disease, provides a stringent clinical test for this combination approach.

## 2. Materials and Methods

### 2.1. Study Population

Fourteen consecutive patients with uHCC treated with LEN–New FP at Saitama Red Cross Hospital between April 2022 and March 2025 were retrospectively analyzed. All patients were aged ≥20 years and had radiologically or histologically confirmed HCC. Unresectability was determined by a multidisciplinary tumor board based on tumor extent, vascular invasion, hepatic reserve, and technical feasibility. Patients with bilobar disease, extensive intrahepatic tumor burden, or PVTT (including Vp3 and Vp4) were eligible. Primary ICI resistance was defined as disease progression (per mRECIST) at the first scheduled imaging assessment following initiation of an ICI-containing regimen, without achieving any response or stable disease.

All 14 patients were included in the clinical outcome analyses. PHA diameter measurement was feasible in 13 patients; 1 patient was excluded from vascular diameter analysis due to insufficient angiographic visualization.

### 2.2. Catheter Placement

A Shepherd hook-type 4-French catheter was inserted into the femoral artery via a 4-French introducer sheath using the Seldinger technique and advanced to the target artery under fluoroscopy. Two techniques were used for port-catheter placement, selected at the operator’s discretion based on vascular anatomy and technical considerations. In most cases, the right gastric artery and gastroduodenal artery were embolized with microcoils (Target 360 XL/XXL, Stryker; Ruby Coil/POD Packing Coil, Penumbra; or VortX Diamond Shape Fibered Platinum Coils/VortX Fibered Platinum Coils, Boston Scientific) to fix the catheter tip and prevent gastroduodenal toxicity from intra-arterially delivered chemotherapeutic agents (GDA coiling method). In selected cases where anatomical conditions precluded coil embolization, the catheter tip was placed directly into the hepatic artery without coil fixation (direct placement method). Hemodynamic modification of collateral arteries (e.g., right inferior phrenic artery) was performed as needed for tumors with extrahepatic arterial supply or aberrant vasculature.

A polyurethane-coated indwelling catheter (Anthron P-U catheter, Toray Medical, Tokyo, Japan; or PIOLAX W Spiral Catheter, PIOLAX, Kanagawa, Japan) was placed with its tip in the common or proper hepatic artery and connected to a subcutaneously implanted injection port.

### 2.3. Treatment Protocol

Lenvatinib was initiated 14–28 days after catheter placement and administered orally at 12 mg/day for patients weighing ≥60 kg or 8 mg/day for those weighing <60 kg [4]. Lenvatinib was withheld two days before and resumed two days after each HAIC session. Dose reductions were permitted at the attending physician’s discretion based on adverse events, performance status, and patient age.

The New FP HAIC regimen consisted of cisplatin (50 mg) suspended in 5–10 mL of lipiodol administered intra-arterially, followed immediately by an initial 5-FU bolus (250 mg), and then continuous 5-FU infusion (1250 mg) over the subsequent five days via a balloon pump (SUREFUSER PUMP; Nipro Pharma Corporation, Osaka, Japan), for a total 5-FU dose of 1500 mg over five days [19,20]. Cycles were repeated every two to four weeks in the outpatient setting for as long as possible. The cisplatin dose was adjusted at each session based on angiographic findings.

**Figure 1:**
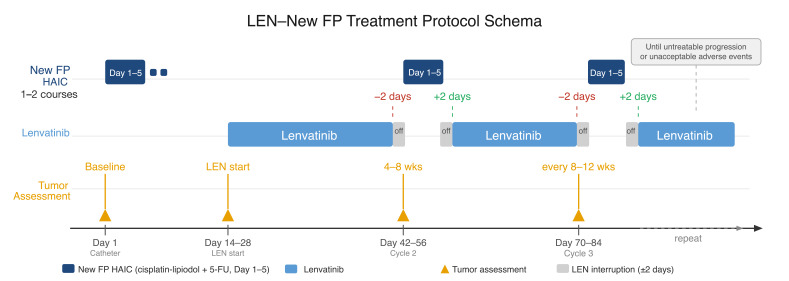
LEN–New FP treatment protocol schema. New FP HAIC (cisplatin–lipiodol plus continuous 5-fluorouracil infusion, Days 1–5; dark blue) was initiated first, followed by lenvatinib (light blue) starting on Days 14–28 after catheter placement. Lenvatinib was discontinued two days before each New FP cycle and resumed two days after its completion (gray segments). New FP cycles were repeated every 2–4 weeks until untreatable progression or unacceptable adverse events. Tumor assessment (yellow arrows) was performed at baseline, at lenvatinib initiation, at 4–8 weeks after the first New FP cycle, and every 8–12 weeks thereafter. Abbreviations: HAIC, hepatic arterial infusion chemotherapy; LEN, lenvatinib; New FP, cisplatin–lipiodol plus continuous 5-fluorouracil infusion; wks, weeks.

Tumor response was assessed on contrast-enhanced CT at baseline and every 4–8 weeks thereafter, with subsequent evaluations every 8–12 weeks. The overall treatment schema is illustrated in Figure 1.

### 2.4. Imaging Assessment and Arterial Diameter Measurement

Tumor response was assessed using mRECIST criteria [30]. PHA diameter was measured on angiographic images obtained during HAIC procedures at treatment initiation and at the time of best radiological response. Measurements were performed at a predefined, reproducible segment of the PHA using an identical angiographic projection whenever feasible. Two independent hepatologists performed all measurements using calibrated imaging software.

In addition to PHA diameter measurement, tumor vascularity was semi-quantitatively assessed on angiographic images at treatment initiation and at best radiological response, using a 4-point visual scoring system (Tumor Vascularity Score [TVS]): Score 0, no apparent tumor stain or tumor-feeding vessels; Score 1, mild tumor stain or sparse tumor-feeding vessels; Score 2, moderate tumor stain or visible tumor-feeding vessels; Score 3, marked tumor stain with prominent tumor-feeding vessels. This assessment was performed by two independent hepatologists and was intended as an exploratory complement to PHA diameter measurements. In patients with multiple hepatic lesions, the worst TVS among all evaluable lesions was assigned as the representative score at each time point.

### 2.5. Outcome Measures

The primary endpoint was ORR based on mRECIST [30]. ORR was defined as the proportion of patients achieving a complete response (CR) or partial response (PR). DCR was defined as the proportion of patients achieving CR, PR, or stable disease (SD). Secondary endpoints included DCR, change in PHA diameter, conversion to curative-intent therapy [31], OS from LEN–New FP initiation (assessed through September 2025), iPFS, and treatment safety assessed by CTCAE version 5.0. iPFS was defined as the time from LEN–New FP initiation to the first occurrence of intrahepatic disease progression per mRECIST, or death from any cause, whichever occurred first. Patients who underwent conversion to curative-intent therapy (surgical resection or ablation) without prior documented progression were censored at the time of the curative procedure, as the intent of treatment had shifted from disease control to cure. Patients without events were censored at the last imaging assessment.

### 2.6. Ethical Approval

This retrospective study was approved by the Institutional Review Board of Saitama Red Cross Hospital (IRB No. 24-J; approval date: 19 September 2024) and conducted in accordance with the Declaration of Helsinki and the STROBE reporting guidelines [32].

Patient data were retrospectively collected from clinical records, and no interventions were performed for research purposes.

The requirement for informed consent was waived due to the retrospective nature of the study, and an opt-out approach was implemented in accordance with institutional guidelines.

## 3. Results

### 3.1. Patient Characteristics

The baseline characteristics of the 14 patients are summarized in Table 1. The median age was 71.0 years (range: 53–80). Most patients were male (*n* = 12, 85.7%), and the predominant etiology was non-viral liver disease (*n* = 10, 71.4%). Thirteen patients (92.9%) had received prior ICI-containing systemic therapy and had discontinued treatment due to refractoriness or intolerance; primary ICI resistance was observed in ten patients (71.4%). All 14 patients exceeded the Up-to-7 and Up-to-11 criteria, and macrovascular invasion was present in 9 patients (64.3%). Extrahepatic spread was present in three patients (21.4%). Only one patient received LEN–New FP as first-line therapy.

### 3.2. Survival Outcomes and Treatment Continuity

**Figure 2:**
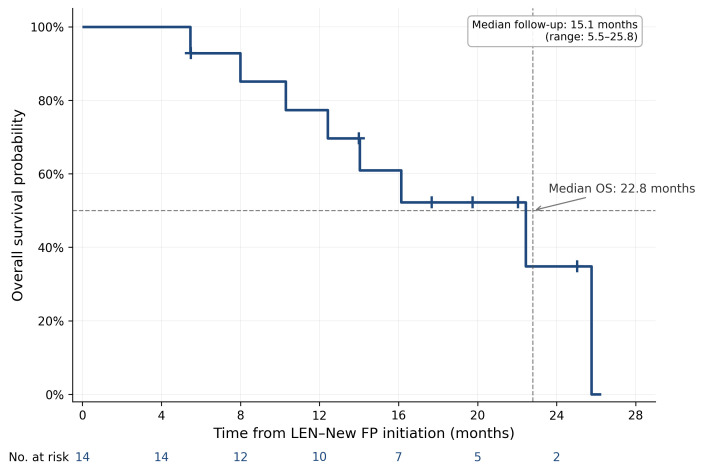
Overall survival from LEN–New FP initiation. Kaplan–Meier curve showing overall survival (OS) from the initiation of LEN–New FP therapy in all 14 patients. The x-axis represents time from LEN–New FP initiation in months; the y-axis represents the probability of overall survival. Median OS was 22.8 months at a median follow-up of 15.1 months (range: 5.5–25.8 months). The dashed horizontal line indicates the 50% survival probability used to determine median OS; the dashed vertical line indicates the corresponding median OS time point. Tick marks on the survival curve indicate censored observations. The number of patients at risk at each time point is shown below the x-axis. OS was defined as the time from LEN–New FP initiation to death from any cause; patients alive at the last follow-up were censored.

**Figure 3:**
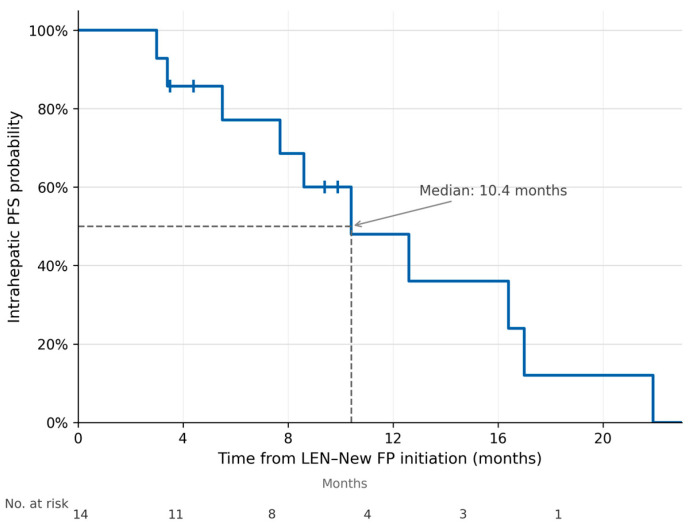
Intrahepatic progression-free survival (iPFS). Kaplan–Meier curve showing iPFS from LEN–New FP initiation. iPFS was defined as time to intrahepatic progression (per mRECIST) or death; patients achieving conversion to curative-intent therapy without prior progression were censored at the time of the curative procedure. Median iPFS was 10.4 months. Tick marks indicate censored observations.

**Figure 4:**
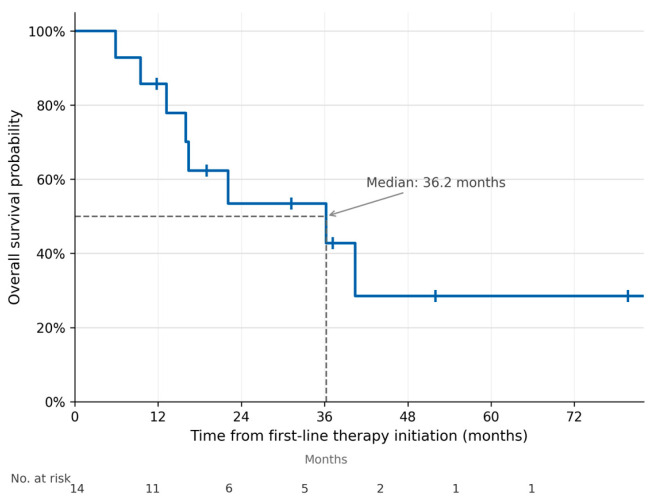
Overall survival from initiation of first-line therapy. Kaplan–Meier curve showing OS from the start of first-line systemic therapy in all 14 patients. Median OS from first-line initiation was 36.2 months at a median follow-up of 20.6 months (range: 5.9–79.7). Tick marks indicate censored observations.

The median OS from LEN–New FP initiation was 22.8 months (assessed through September 2025), and the median iPFS was 10.4 months (Figure 2 and Figure 3). The median follow-up duration from LEN–New FP initiation was 15.1 months (range: 5.5–25.8 months). When measured from the initiation of first-line systemic therapy, the median OS was 36.2 months at a median follow-up of 20.6 months (range: 5.9–79.7 months), reflecting the contribution of sequential treatment strategies including LEN–New FP (Figure 4).

A total of 11 of 14 patients (78.6%) were able to transition to subsequent therapies, including additional systemic therapy, locoregional treatment, or conversion to curative-intent therapy (*n* = 4). This high transition rate reflects preservation of hepatic reserve and sustained treatment feasibility during LEN–New FP therapy. Treatment-related adverse events (TRAEs) are summarized in Table 2. The most common TRAEs of any grade were hypertension, fatigue, and decreased appetite (each 64.3%), followed by hypothyroidism, and weight loss (each 42.9%). Grade 3–4 TRAEs occurred in 8 patients (57.1%) and included cisplatin-induced anaphylaxis (*n* = 3, 21.4%), implant infection (*n* = 3, 21.4%), hypertension (*n* = 2, 14.3%), biloma (*n* = 2, 14.3%), decreased appetite (*n* = 1, 7.1%), and hypothyroidism (*n* = 1, 7.1%). No cases of hepatic failure or treatment-related death occurred. Lenvatinib dose reduction was required in 12 of 14 patients (85.7%), primarily due to hypertension, fatigue, and appetite loss, yet antitumor efficacy was maintained throughout.

### 3.3. Hepatic Artery Remodeling During LEN–New FP Therapy

Changes in PHA diameter were assessed using angiographic images obtained at LEN–New FP initiation and at the time of best radiological response. PHA diameter measurement was feasible in 13 of 14 patients; one patient was excluded due to inadequate angiographic visualization.

**Figure 5:**
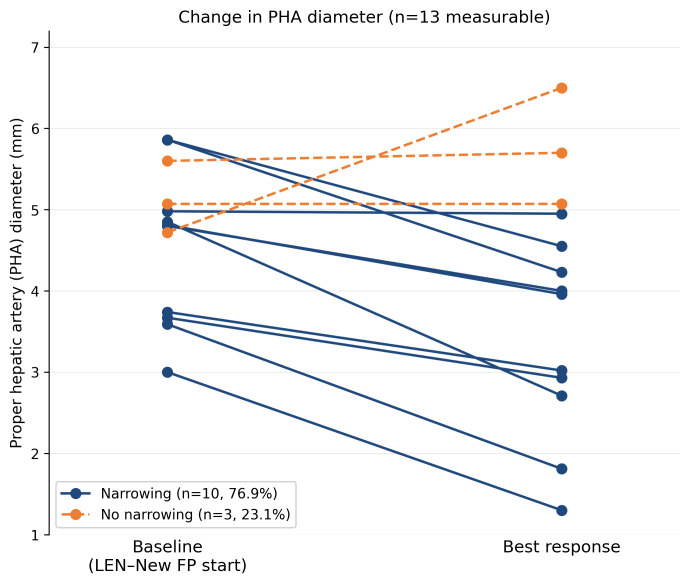
Changes in proper hepatic artery (PHA) diameter during LEN–New FP therapy. Paired plot showing PHA diameter (mm) measured on angiography at LEN–New FP initiation and at the time of best radiological response in 13 evaluable patients. Each line represents one patient. PHA narrowing was observed in 10 of 13 patients (76.9%), with a median reduction of 1.08 mm (21.3%). Patients receiving higher lenvatinib dose intensity tended to exhibit more pronounced arterial narrowing, whereas attenuation of narrowing was observed after dose reduction. No clear association was identified between PHA narrowing and deterioration of hepatic function.

**Figure 6:**
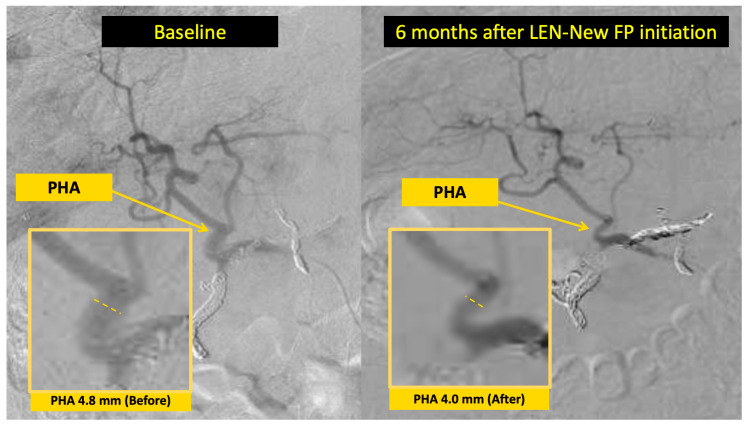
Representative angiographic images of proper hepatic artery (PHA) narrowing in Case 4. Hepatic angiography performed at baseline (**left**) and 6 months after LEN–New FP initiation (**right**) demonstrates progressive reduction in PHA caliber from 4.8 mm to 4.0 mm (16.7% reduction). This angiographic finding is representative of the PHA narrowing pattern observed during LEN–New FP therapy. PHA, proper hepatic artery; LEN–New FP, lenvatinib plus New FP hepatic arterial infusion chemotherapy.

**Figure 7:**
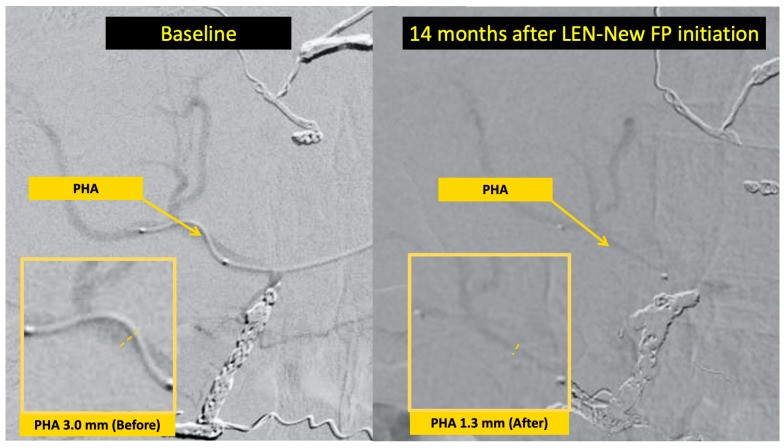
Representative angiographic images of marked proper hepatic artery (PHA) narrowing in Case 2. Hepatic angiography performed at baseline (**left**) and 14 months after LEN–New FP initiation (**right**) demonstrates marked reduction in PHA caliber from 3.0 mm to 1.3 mm (56.7% reduction), representing one of the most pronounced cases of arterial narrowing in this cohort. This degree of narrowing was observed in a patient receiving 8 mg of lenvatinib on alternate days and achieving a partial response. PHA, proper hepatic artery; LEN–New FP, lenvatinib plus New FP hepatic arterial infusion chemotherapy.

Progressive PHA narrowing between treatment initiation and best response was observed in 10 of 13 evaluable patients (76.9%), with a median reduction of 1.08 mm (21.3%) (Figure 5). Representative angiographic images illustrating moderate narrowing (Case 4; 4.8 to 4.0 mm, −16.7%) and marked narrowing (Case 2; 3.0 to 1.3 mm, −56.7%) are shown in Figure 6 and Figure 7, respectively.

**Figure 8:**
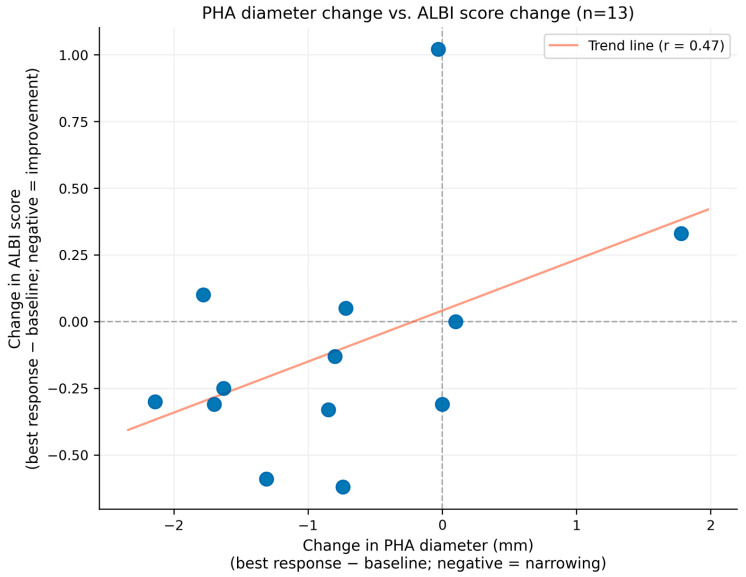
Scatter plot showing the relationship between change in proper hepatic artery (PHA) diameter and change in ALBI score at the time of best radiological response (*n* = 13 evaluable patients). The x-axis represents the change in PHA diameter (mm; best response minus baseline), where negative values indicate arterial narrowing. The y-axis represents the change in ALBI score (best response minus baseline), where negative values indicate improvement in hepatic function. No significant correlation was observed between the degree of PHA narrowing and change in ALBI score (Pearson r = 0.465, *p* = 0.109), supporting the absence of clinically meaningful hepatic ischemia related to arterial caliber reduction. PHA, proper hepatic artery; ALBI, albumin–bilirubin; LEN–New FP, lenvatinib plus New FP hepatic arterial infusion chemotherapy. Note: ALBI score (continuous) was used for correlation analysis; mALBI grade classification was used for clinical outcome assessment.

No clear association was identified between the degree of PHA narrowing and deterioration of hepatic function. PHA diameter changes did not correlate with worsening mALBI grade; at the time of the best radiological response, the mALBI grade improved in 4 patients (28.6%), remained stable in 6 (42.9%), and worsened in 4 (28.6%), indicating overall preservation of hepatic reserve during treatment (Figure 8).

When PHA diameter changes were examined in relation to lenvatinib dose intensity, a trend toward more pronounced arterial narrowing was observed in patients receiving higher lenvatinib doses; conversely, attenuation of PHA narrowing appeared to occur after dose reduction. Although these observations were based on a limited sample size and were not subjected to formal statistical testing, they suggest a possible dose-dependent effect of lenvatinib on hepatic arterial caliber.

Notably, despite dose reductions in the majority of patients (12 of 14 patients [85.7%] required dose reduction to 8 mg every other day [*n* = 8, 57.1%] or 4 mg every other day [*n* = 4, 28.6%]), the antitumor efficacy of LEN–New FP was maintained, with an ORR of 85.7% and DCR of 100% across the cohort. Only one patient received full-dose lenvatinib (12 mg/day) throughout without dose reduction. This finding suggests that the dose of lenvatinib required to potentiate HAIC-mediated tumor control may be substantially lower than that required for lenvatinib monotherapy to achieve direct tumor shrinkage.

**Figure 9:**
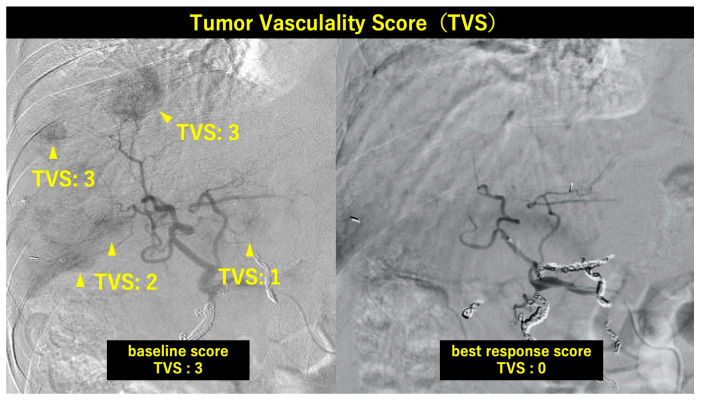
Digital subtraction angiography (DSA) images obtained at baseline (**left**) and at best radiological response (**right**) are shown for a representative patient with multiple intrahepatic lesions. At baseline, individual lesions demonstrate variable degrees of tumor vascularity (TVS 1, 2, and 3, indicated by arrowheads), reflecting the heterogeneity of tumor-feeding vessel architecture across lesions. The worst score among all evaluable lesions (TVS 3: marked tumor staining with prominent tumor-feeding vessels) was assigned as the representative baseline TVS. At best radiological response, tumor staining and tumor-feeding vessels are no longer apparent in any lesion, yielding a representative TVS of 0. This approach—selecting the worst lesion score at each time point—was applied consistently across all patients to avoid underestimation of tumor vascularity.

As a complementary exploratory analysis, tumor vascularity was assessed using the TVS system on paired angiographic images. Among 12 patients with baseline TVS > 0 (two patients had TVS = 0 at baseline and were excluded), TVS decreased in 10 (83.3%), with a median reduction of 1 point. No patient showed an increase in TVS. A directional association was observed between TVS reduction and objective tumor response: TVS decreased in 9 of 10 PR/CR patients with evaluable baseline TVS (90.0%), compared with 1 of 2 SD patients. A modest directional correlation was observed between PHA diameter change and TVS change (Spearman r = −0.541, *p* = 0.056), though formal statistical conclusions are limited by sample size. These findings are consistent with the angiographic observations reported by Tachiiri et al. [28], who demonstrated lenvatinib-induced normalization of tumor neovasculature directly visualized on DSA (Figure 9).

## 4. Discussion

The present study provides the first systematic evaluation of LEN–New FP in a cohort enriched for aggressive disease phenotypes, including patients with ICI-refractory or -intolerant uHCC, extensive tumor burden, and macrovascular invasion—populations with historically limited therapeutic options. The regimen demonstrated substantial antitumor activity across multiple endpoints, with an ORR of 85.7%, a DCR of 100%, and a median OS of 22.8 months from treatment initiation, outcomes that compare favorably with historical data in this challenging patient population.

A key methodological consideration in interpreting the angiographic findings of this study relates to the primary target of anti-VEGF-mediated vascular effects. Vascular normalization induced by VEGFR inhibitors predominantly affects tumor neovasculature—the disordered, immature vessels supplying the tumor—rather than structurally normal proximal arteries such as the PHA [25,26]. Accordingly, the progressive PHA narrowing observed in this cohort is unlikely to represent direct vascular normalization of tumor vessels per se; rather, it may reflect a combination of hemodynamic redistribution following tumor burden reduction, VEGFR inhibition-mediated suppression of endothelial nitric oxide production, and cumulative intimal remodeling from repeated intra-arterial chemotherapy. To complement PHA diameter measurements with more direct evidence of tumor-level vascular change, we additionally assessed tumor vascularity using the TVS system, which demonstrated reduction in 83.3% of evaluable patients and a directional association with objective response [28].

In this study, LEN–New FP achieved sustained intrahepatic disease control and encouraging survival outcomes in patients with advanced uHCC, predominantly when administered as second-line or later therapy. By integrating HAIC with systemic lenvatinib, this regimen provided both potent local tumor control and systemic disease management, translating into substantial clinical outcomes despite aggressive tumor biology. Importantly, no established therapeutic option currently exists for patients who are refractory or intolerant to ICI-based regimens, and the present results suggest that LEN–New FP may address this unmet clinical need.

Intrahepatic disease control emerged as a central contributor to prolonged survival. Although the cohort was characterized by extensive tumor burden, frequent macrovascular invasion, and a high prevalence of ICI refractoriness or intolerance, LEN–New FP achieved sustained suppression of intrahepatic disease progression. This is clinically important because liver failure, rather than extrahepatic progression, is frequently the proximate cause of death in advanced HCC. This sustained intrahepatic tumor control highlights the therapeutic value of combining locoregional interventional approaches with systemic therapy in this difficult-to-treat population.

Treatment feasibility and continuity were further supported by the high rate of transition to subsequent therapies. In our cohort, 78.6% of patients were able to proceed to additional treatment modalities, including conversion therapy, further systemic therapy, or locoregional interventions. This high transition rate suggests preservation of hepatic reserve during LEN–New FP therapy, which is essential for maintaining sequential treatment options in advanced HCC. Of note, only one patient received LEN–New FP as first-line therapy, indicating that this regimen functions primarily as an effective subsequent-line strategy rather than a replacement for established first-line approaches [4,5,6,7,8,11,23,33].

Mechanistically noteworthy is the finding that robust antitumor efficacy was maintained despite lenvatinib dose reductions in 12 of 14 patients (85.7%). Lenvatinib as monotherapy is known to exert dose-dependent antitumor effects, and dose reductions are frequently associated with disease progression in the monotherapy setting, sometimes compelling patients to tolerate significant toxicity in order to preserve efficacy [4,21]. In contrast, our results suggest that within the LEN–New FP combination framework, a substantially lower lenvatinib dose may be sufficient to sustain therapeutic synergy with HAIC. We hypothesize that the dose threshold required for lenvatinib to achieve direct tumor regression as monotherapy differs fundamentally from the dose required to exert vascular normalization and VEGF inhibition sufficient to potentiate HAIC-mediated cytotoxicity. Even at reduced doses, lenvatinib may preserve its capacity to normalize tumor vasculature, reduce interstitial pressure, and enhance intratumoral drug delivery—effects that are central to the synergistic mechanism of LEN–New FP [25,26,27]. This dissociation between the dose required for monotherapy efficacy and the dose required for combination synergy represents a meaningful paradigm shift. In conventional lenvatinib monotherapy, patients and physicians often face a difficult trade-off between maintaining adequate drug exposure and managing adverse events. In the LEN–New FP regimen, proactive dose reduction appears feasible without compromising tumor control, potentially reducing the physical, economic, and psychological burden on patients. This tolerability advantage may be particularly valuable in the real-world setting, where many patients with advanced HCC have compromised hepatic reserve and limited capacity to tolerate prolonged high-intensity systemic therapy.

A potential association between lenvatinib dose intensity, PHA narrowing, and treatment efficacy warrants consideration. The dose-dependent nature of VEGFR inhibition suggests that higher lenvatinib exposure may result in more pronounced endothelial NO suppression and, consequently, greater hepatic arterial narrowing. At the same time, dose reductions—driven by adverse events or tolerability concerns—may attenuate this vascular effect. Importantly, the present cohort was enriched with patients meeting the Niizeki criteria for poor prognosis with New FP monotherapy [19,21], representing a disease population inherently refractory to HAIC alone. This case composition may have amplified the apparent correlation between lenvatinib dose and therapeutic outcomes, as New FP alone would have been insufficient for disease control in the majority of cases. Notably, five patients in this cohort had previously received lenvatinib monotherapy and experienced progressive disease on that regimen; all five responded to LEN–New FP, suggesting that the addition of New FP—including its embolic and cytotoxic components—is essential to therapeutic efficacy and that the synergistic interaction between lenvatinib and New FP extends beyond what either agent achieves alone. This finding underscores both the limitations of lenvatinib monotherapy and the mechanistic rationale for combination with intra-arterial chemotherapy.

The high ORR and DCR are especially striking given the unfavorable prognostic features of the cohort. This pattern is consistent with emerging evidence that integration of locoregional and systemic therapy can overcome resistance mechanisms and enhance antitumor efficacy. A plausible mechanistic basis for this synergy is lenvatinib-induced vascular remodeling, which may improve intratumoral drug delivery and potentiate the cytotoxic effects of HAIC [4,18,19,20,25,26,27].

The most clinically striking finding in this study was the progressive narrowing of the PHA observed on angiography during LEN–New FP therapy. To our knowledge, this is the first report to directly evaluate angiographic changes in PHA diameter under a lenvatinib-containing HAIC combination. Notably, PHA diameter was not a prespecified endpoint but an incidental finding from routine angiography; accordingly, we do not propose it as a novel biomarker.

Nevertheless, this angiographic observation provides a compelling mechanistic insight that may help explain the exceptionally high tumor response rate achieved with LEN–New FP. HAIC alone is empirically known to induce arterial narrowing with repeated treatment sessions. The degree of PHA narrowing appeared to be further influenced by lenvatinib dose intensity, with more pronounced changes at higher doses and partial attenuation after dose reduction. Although exploratory, this suggests a dose-dependent contribution of lenvatinib to arterial caliber changes. Lenvatinib also exerts potent VEGF receptor inhibition, influencing vascular tone and endothelial stability—clinically reflected by adverse events such as hypertension [4]. Lenvatinib-associated hypertension is mechanistically attributed to VEGFR2 inhibition-mediated suppression of endothelial nitric oxide (NO) synthase activity, leading to reduced NO production, impaired vasodilation, and increased peripheral vascular resistance [34,35]. We hypothesize that an analogous mechanism may operate at the level of the proper hepatic artery: VEGFR inhibition-induced reduction in NO-mediated vasodilation in the hepatic arterial endothelium may contribute to the progressive narrowing of PHA caliber observed in this cohort, independent of—or in addition to—tumor burden reduction. This hypothesis is consistent with the observation that PHA narrowing was evident, even in patients without marked tumor shrinkage, and is further supported by the occurrence of hepatic artery occlusion in one patient, suggesting that lenvatinib may exert direct arterial wall effects beyond those attributable to hemodynamic redistribution alone. Prospective studies incorporating endothelial function biomarkers and serial hemodynamic measurements would be needed to formally test this hypothesis. When combined, HAIC and lenvatinib may cooperatively promote PHA narrowing, thereby reducing the tumor vascular bed, increasing local chemotherapeutic concentrations, and enhancing the embolic effect of lipiodol deposition [19,20,29]. These processes may establish a positive feedback loop amplifying intrahepatic antitumor efficacy.

Interestingly, no clear association was observed between PHA narrowing and deterioration of hepatic function. Despite angiographic evidence of arterial caliber reduction, hepatic function was preserved or improved in many patients at the time of best radiological response. This dissociation suggests that angiographically observed PHA narrowing does not necessarily indicate clinically significant hepatic ischemia; rather, it may reflect favorable vascular remodeling accompanying tumor burden reduction and improved intrahepatic hemodynamics [19,20,25,26,27]. Consistent with our angiographic findings, a Chinese retrospective study reported that hepatic artery diameters measured on contrast-enhanced CT decreased rapidly after FOLFOX-HAIC plus lenvatinib, and that shrinkage of the tumor-feeding artery correlated with improved short-term efficacy [36]. Collectively, these findings reinforce the mechanistic plausibility of LEN–New FP within the framework of vascular normalization and highlight the potential role of vascular dynamics in immuno-oncology–systemic combination therapy [18,23,25,26,27,36].

However, the progressive narrowing of the PHA observed during LEN–New FP therapy may carry a dual significance. While arterial narrowing appears to correlate with tumor response, it may also represent a potential adverse vascular effect with clinical consequences over repeated treatment cycles. In our cohort, biloma (Grade 3) was observed as a treatment-related adverse event in two patients, and biliary complications following prolonged HAIC have been reported in the literature [37]. We hypothesize that cumulative PHA narrowing may lead to peribiliary arterial injury—damage to the small arterioles supplying the bile ducts—ultimately predisposing to biliary ischemia and biloma formation. This mechanistic pathway warrants careful monitoring of biliary complications in patients receiving long-term LEN–New FP therapy.

Cisplatin-induced anaphylaxis occurred in 3 of 14 patients (21.4%), all developing at or after the 8th cycle of New FP HAIC. This incidence appears higher than the reported frequency of cisplatin hypersensitivity reactions following intravenous administration, which ranges from approximately 5–20% for all grades, with severe (Grade 3–4) reactions reported in fewer than 5% of patients [38,39]. Although corticosteroid premedication has been widely used in clinical practice, evidence suggests that steroid prophylaxis offers limited efficacy against true platinum-induced anaphylaxis; in contrast to iodinated contrast media hypersensitivity, IgE-mediated type I reactions to cisplatin are not reliably prevented by corticosteroids [39,40]. The higher incidence observed in our cohort may reflect the unique pharmacokinetic characteristics of intra-arterial administration: manual injection of cisplatin suspended in lipiodol under fluoroscopy may result in a rapid and concentrated bolus delivery to the hepatic circulation, potentially triggering abrupt mast cell degranulation at higher local drug concentrations than achievable with conventional intravenous infusion. Potential countermeasures include substituting lipiodol alone (omitting cisplatin) from the eighth cycle onward, replacing cisplatin with epirubicin, or reducing the manual injection rate to minimize peak arterial concentrations. Complete prevention of cisplatin-induced anaphylaxis in this setting remains challenging and warrants further prospective evaluation. Awareness of this risk is particularly important given the cumulative nature of platinum sensitization in patients receiving repeated HAIC cycles.

The dissociation between PHA narrowing and hepatic functional deterioration observed in our cohort also merits mechanistic consideration. Although it is theoretically plausible that progressive reduction in PHA caliber could impair hepatic perfusion, our data did not support a clinically meaningful association between the degree of PHA narrowing and changes in ALBI score (Pearson r = 0.465, *p* = 0.109). This finding may be explained by at least two compensatory mechanisms. First, collateral hepatic arterial supply—including the development of extrahepatic arterial pathways from the inferior phrenic artery and other perihepatic vessels—may partly offset the reduction in PHA flow. Second, and perhaps more importantly, the substantial reduction in intrahepatic tumor burden achieved with LEN–New FP therapy may itself improve hepatic function by reducing tumor-mediated hepatocellular compression and intratumoral arteriovenous shunting. This net improvement in residual liver function may obscure or counterbalance any adverse hemodynamic effect of PHA narrowing, and should be considered when interpreting the vascular and functional findings together.

LEN–New FP and LEN–TACE represent complementary but distinct endovascular strategies within the evolving landscape of HCC combination therapy. LEN–TACE trials, including TACTICS-L and the LAUNCH trial, applied relatively restrictive eligibility criteria—excluding tumors >10 cm, >10 lesions, vascular invasion, or extrahepatic metastases—reflecting patient populations close to the up-to-seven criteria considered suitable for TACE [24]. In contrast, the present cohort included patients with extensive tumor burden, macrovascular invasion, and ICI-refractory disease—characteristics that would have rendered the majority ineligible for enrollment in those trials. Despite this unfavorable patient profile, LEN–New FP achieved a high ORR and disease control rate, suggesting a complementary role for HAIC-based combination therapy in patients for whom TACE is unsuitable [21].

From a mechanistic standpoint, the New FP regimen incorporates lipiodol, which possesses a weak embolic effect and the capacity to remain within the tumor vasculature [20,29]. Combined with lenvatinib-induced reduction in the tumor vascular bed, this property may enhance intratumoral drug retention and contribute to the synergistic antitumor effect observed with LEN–New FP. Furthermore, compared with LEN–TACE, LEN–New FP offers practical advantages including outpatient administration, lower cost, ease of repetition, and applicability in patients with high tumor burden or vascular invasion in whom TACE is technically difficult or oncologically inappropriate [21].

**Figure 10:**
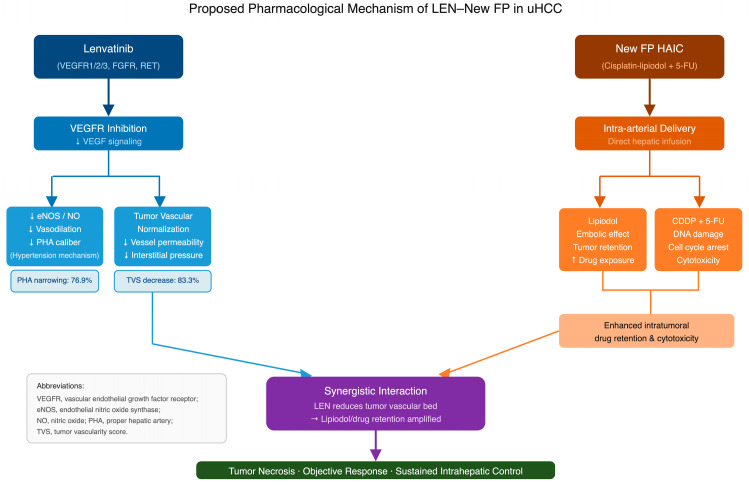
Proposed pharmacological mechanism of LEN–New FP in unresectable hepatocellular carcinoma. Lenvatinib exerts dual vascular effects through VEGFR inhibition: suppression of endothelial nitric oxide synthase (eNOS) activity leads to reduced nitric oxide (NO) production and impaired vasodilation, contributing to proper hepatic artery (PHA) narrowing (observed in 76.9% of evaluable patients); and normalization of tumor neovasculature reduces vascular permeability and intratumoral interstitial pressure. New FP HAIC delivers cisplatin–lipiodol and 5-fluorouracil directly into the hepatic artery; lipiodol provides an embolic effect with prolonged intratumoral retention, while cisplatin and 5-FU exert direct cytotoxicity. The synergistic interaction between these two pathways—whereby lenvatinib-induced reduction in the tumor vascular bed amplifies lipiodol and drug retention—is proposed as the mechanistic basis for the high antitumor efficacy of LEN–New FP, ultimately resulting in tumor necrosis, objective response, and sustained intrahepatic disease control. Clinical angiographic observations from this cohort, including PHA narrowing (76.9%) and tumor vascularity score reduction (83.3%), are incorporated as supporting evidence. Abbreviations: eNOS, endothelial nitric oxide synthase; FGFR, fibroblast growth factor receptor; HAIC, hepatic arterial infusion chemotherapy; LEN, lenvatinib; NO, nitric oxide; PHA, proper hepatic artery; RET, rearranged during transfection proto-oncogene; TVS, tumor vascularity score; VEGFR, vascular endothelial growth factor receptor; 5-FU, 5-fluorouracil; CDDP, cisplatin.

Taken together, LEN–New FP represents a valuable component of multimodal treatment strategies for advanced HCC, particularly in patients with ICI-refractory or -intolerant disease, extensive intrahepatic tumor burden, and macrovascular invasion—populations with historically limited therapeutic options [10,11]. By achieving sustained intrahepatic control, maintaining treatment feasibility, and enabling further therapeutic interventions, LEN–New FP may contribute to prolonged survival across the disease course. The incidental yet consistent observation of PHA narrowing provides a mechanistic rationale for the remarkable antitumor efficacy of this regimen and underscores the potential importance of vascular dynamics in immuno-oncology–systemic combination therapy [18,23,25,26,27,36]. The proposed pharmacological mechanisms underlying LEN–New FP efficacy, integrating the vascular effects of lenvatinib and the cytotoxic–embolic effects of New FP, are schematically summarized in Figure 10. The broader framework of systemic therapy combined with locoregional therapy, within which LEN–New FP is positioned, has been comprehensively reviewed by Kudo [41], who highlighted lenvatinib-induced vascular normalization—including reduction in vascular permeability and intratumoral interstitial pressure—as a key mechanistic rationale for the synergistic efficacy of lenvatinib–locoregional therapy combinations. Although these findings are exploratory and derived from a retrospective cohort, prospective studies are warranted to confirm these observations and to refine optimal patient selection and treatment positioning within evolving algorithms [18,23,33].

## 5. Limitations

This study has several limitations. First, it was a single-center retrospective analysis with a limited sample size of 14 patients, which restricts the generalizability of the findings. Second, PHA diameter assessment was feasible in only 13 patients, and vascular measurements were not standardized prospectively. Third, the retrospective design introduces potential selection bias, particularly regarding patients deemed suitable for catheter-based therapy and combination treatment. Fourth, heterogeneity in prior treatment lines and lenvatinib dose modifications may have influenced clinical outcomes. Fifth, the absence of a control group precludes direct comparison with alternative treatment strategies. Sixth, the Tumor Vascularity Score (TVS) is a semi-quantitative and inherently subjective measure; although scoring was performed by two independent hepatologists, inter-observer variability was not formally assessed. Furthermore, TVS reduction may reflect tumor burden reduction rather than true vascular normalization, and these findings should be interpreted as hypothesis-generating rather than confirmatory. Additionally, the censoring of patients who underwent curative-intent conversion at the time of their procedure may represent a form of informative censoring, as these patients were by definition responding to therapy. As a sensitivity analysis, when these four patients were treated as events (i.e., censored at the time of progression imputed as the curative procedure date) rather than censored, the median iPFS remained consistent with the primary analysis, supporting the robustness of the reported estimate. The present findings should therefore be interpreted as exploratory and warrant validation in larger prospective studies.

## 6. Conclusions

In this retrospective analysis, lenvatinib combined with New FP HAIC demonstrated potent antitumor activity and acceptable tolerability in patients with aggressive uHCC, including those with ICI-refractory or -intolerant disease and significant macrovascular invasion [10,21]. The high ORR, meaningful conversion-to-curative-therapy rate, and encouraging OS highlight the therapeutic value of this regimen [21,31]. The incidental angiographic observation of PHA narrowing in the majority of patients provides important mechanistic insight into the cooperative interplay between VEGFR inhibition and intra-arterial chemotherapy, offering a compelling rationale for the exceptional antitumor activity observed with this regimen. These findings support LEN–New FP as a promising multimodal strategy within the evolving landscape of immuno-oncology–systemic therapy integration [16,23,24].

## Figures and Tables

**Table 1:** Baseline patient characteristics (*n* = 14).

Characteristic	Value (*n* = 14)
Age, years, median (range)	71.0 (53–80)
Sex, male/female, *n* (%)	12 (85.7)/2 (14.3)
Etiology, *n* (%)	
HBV	1 (7.1)
HCV	3 (21.4)
Non-viral (NASH/alcohol/other)	10 (71.4)
ECOG performance status, *n* (%)	
0	8 (57.1)
1	5 (35.7)
2	1 (7.1)
Child–Pugh class, *n* (%)	
A	13 (92.9)
B	1 (7.1)
mALBI grade, *n* (%)	
Grade 1	5 (35.7)
Grade 2a	4 (28.6)
Grade 2b	5 (35.7)
BCLC stage, *n* (%)	
B	7 (50.0)
C	7 (50.0)
Tumor size, largest lesion, mm, median (range)	55.5 (15–127)
Tumor number, median (range)	Multiple (≥8 nodules or bilobar) in 8; median 3 (range 1–9) in remainder
Beyond Up-to-7 criteria, *n* (%)	14 (100)
Beyond Up-to-11 criteria, *n* (%)	14 (100)
Portal vein tumor thrombus, *n* (%)	
Vp2	1 (7.1)
Vp3	1 (7.1)
Vp4	4 (28.6)
None	5 (35.7)
vv3 (hepatic vein invasion)	2 (14.3)
b4 (bile duct invasion)	1 (7.1)
Macrovascular invasion (any), *n* (%)	9 (64.3)
Extrahepatic spread, *n* (%)	3 (21.4)
AFP, IU/mL, median (range)	151.1 (3.5–20,200)
Prior lines of systemic therapy, *n* (%)	
First-line (LEN–New FP as 1L)	1 (7.1)
Second-line	7 (50.0)
Third-line or later	6 (42.9)
Prior ICI-containing regimen, *n* (%)	13 (92.9)
Primary ICI resistance †, *n* (%)	10 (71.4)
Lenvatinib starting dose ‡, *n* (%)	
8 mg/day (body weight < 60 kg)	0 (0)
12 mg/day (body weight ≥ 60 kg)	2 (14.3)
8 mg every other day (reduced dose)	8 (57.1)
4 mg every other day (further reduced)	4 (28.6)

Abbreviations: HBV, hepatitis B virus; HCV, hepatitis C virus; NASH, non-alcoholic steatohepatitis; ECOG, Eastern Cooperative Oncology Group; mALBI, modified albumin–bilirubin; BCLC, Barcelona Clinic Liver Cancer; AFP, alpha-fetoprotein; ICI, immune checkpoint inhibitor; LEN–New FP, lenvatinib plus New FP hepatic arterial infusion chemotherapy. † Primary ICI resistance: disease progression at first scheduled imaging assessment following ICI-containing therapy initiation, without achieving response or stable disease. ‡ Lenvatinib starting dose reflects the dose at the time of LEN–New FP initiation, not the standard weight-based dosing at first prescription. The majority of patients had undergone prior lenvatinib monotherapy with dose reductions already in place, or were initiated at a reduced dose due to age, performance status, or comorbidities, at the attending physician’s discretion. No patient weighed <60 kg; however, 12 of 14 patients (85.7%) commenced LEN–New FP at a reduced dose (8 mg or 4 mg every other day).

**Table 2:** Treatment-related adverse events (*n* = 14).

Adverse Event	Any Grade *n* (%)	Grade 1–2 *n* (%)	Grade 3–4 *n* (%)
Hypertension	9 (64.3)	7 (50.0)	2 (14.3)
Fatigue	9 (64.3)	9 (64.3)	0
Decreased appetite	9 (64.3)	8 (57.1)	1 (7.1)
Hypothyroidism	6 (42.9)	5 (35.7)	1 (7.1)
Weight loss	6 (42.9)	6 (42.9)	0
Proteinuria	4 (28.6)	4 (28.6)	0
Hyperbilirubinemia	3 (21.4)	3 (21.4)	0
Hypoalbuminemia	3 (21.4)	3 (21.4)	0
Thrombosis	3 (21.4)	3 (21.4)	0
Implant infection	3 (21.4)	0	3 (21.4)
Peptic ulcer	3 (21.4)	3 (21.4)	0
Hand–foot skin reaction	2 (14.3)	2 (14.3)	0
Diarrhea	2 (14.3)	2 (14.3)	0
Hyperthyroidism	2 (14.3)	2 (14.3)	0
Vascular disorders (hepatic artery occlusion)	1 (7.1)	1 (7.1)	0
Alopecia	1 (7.1)	1 (7.1)	0
Biloma	2 (14.3)	0	2 (14.3)
Cisplatin-induced anaphylaxis (immune system disorders)	3 (21.4)	0	3 (21.4)

Adverse events were graded according to the Common Terminology Criteria for Adverse Events (CTCAE) version 5.0.

## Data Availability

The data presented in this study are available on request from the corresponding author.
